# Structural basis for substrate and product recognition in human phosphoglucomutase-1 (PGM1) isoform 2, a member of the α-d-phosphohexomutase superfamily

**DOI:** 10.1038/s41598-020-62548-0

**Published:** 2020-03-27

**Authors:** Paul Hoff Backe, Jon K. Laerdahl, Lene Svendsen Kittelsen, Bjørn Dalhus, Lars Mørkrid, Magnar Bjørås

**Affiliations:** 1 Department of Microbiology, Oslo University Hospital, Oslo, Norway; 2 Department of Medical Biochemistry, Institute for Clinical Medicine, University of Oslo, Oslo, Norway; 3 ELIXIR Norway, Department of Informatics, University of Oslo, Oslo, Norway; 4 Department of Medical Biochemistry, Oslo University Hospital, Oslo, Norway; 5 Department of Microbiology, Institute for Clinical Medicine, University of Oslo, Oslo, Norway; 6 Department of Clinical and Molecular Medicine, Norwegian University of Science and Technology (NTNU), Trondheim, Norway

**Keywords:** Molecular evolution, Glycobiology, Protein sequence analyses, Structural biology, X-ray crystallography

## Abstract

Human phosphoglucomutase 1 (PGM1) is an evolutionary conserved enzyme that belongs to the ubiquitous and ancient α-d-phosphohexomutases, a large enzyme superfamily with members in all three domains of life. PGM1 catalyzes the bi-directional interconversion between α-d-glucose 1-phosphate (G1P) and α-d-glucose 6-phosphate (G6P), a reaction that is essential for normal carbohydrate metabolism and also important in the cytoplasmic biosynthesis of nucleotide sugars needed for glycan biosynthesis. Clinical studies have shown that mutations in the *PGM1* gene may cause PGM1 deficiency, an inborn error of metabolism previously classified as a glycogen storage disease, and PGM1 deficiency was recently also shown to be a congenital disorder of glycosylation. Here we present three crystal structures of the isoform 2 variant of PGM1, both as a free enzyme and in complex with its substrate and product. The structures show the longer N-terminal of this PGM1 variant, and the ligand complex structures reveal for the first time the detailed structural basis for both G1P substrate and G6P product recognition by human PGM1. We also show that *PGM1* and the paralogous gene *PGM5* are the results of a gene duplication event in a common ancestor of jawed vertebrates, and, importantly, that both *PGM1* isoforms are conserved and of functional significance in all vertebrates. Our finding that *PGM1* encodes two equally conserved and functionally important isoforms in the human organism should be taken into account in the evaluation of disease-related missense mutations in patients in the future.

## Introduction

Congenital disorders of glycosylation (CDGs) comprise a group of rare metabolic disorders with deficient glycosylation of proteins and/or lipids^1^. CDGs typically present as multi-systemic disorders with a wide range of clinical manifestations and a large variation in the severity of symptoms, ranging from a mild presentation in adults to severe multi-organ dysfunctions causing infantile mortality. The group constitutes a continuously expanding spectrum of diseases, mainly due to an increasing use of molecular biology techniques (e.g. gene sequencing) for cases presenting with symptoms that could indicate an inborn error of metabolism. The application of modern analytic techniques on disease markers may also reveal new metabolic pathways and interrelations for clinical pictures that hitherto have not been well understood. This may also lead to reclassification of disorders, as recently was the case with phosphoglucomutase 1 (PGM1) deficiency, an autosomal recessive disease which previously was known to be a glycogen storage disease type XIV^2^. It was shown that PGM1 deficiency is also a congenital disorder of protein N-glycosylation, PGM1-CDG^3^. Patients with PGM1-CDG have been reported to have hormonal dysregulation, hepatopathy with elevated liver enzymes, congenital malformations, and dilated cardiomyopathy resulting in severe cases in cardiac arrest. The majority of the patients presents with hypoglycemia and muscle symptoms, including exercise intolerance, muscle weakness and rhabdomyolysis^3,4^. Recently central nervous system involvement was added to the list^5^. As one of very few CDGs, PGM1-CDG is treatable, with d-galactose supplementation mitigating several clinical features as well as restoring protein N-glycosylation^3,6^.

The physiologically important protein PGM1 is an evolutionary conserved enzyme that belongs to the ubiquitous and ancient α-d-phosphohexomutase superfamily, and it is encoded by the *PGM1* gene^7–9^. In humans, there are at least five different α-d-phosphoglucomutase (PGM) isozymes^7,9^ where PGM1 is the most prominent, representing about 90% of total PGM activity (EC 5.4.2.2) in most cell types^10^. PGM1 is a key regulator of carbohydrate metabolism in mammalian cells, catalyzing the reversible conversion between α-d-glucose 1-phosphate (G1P) and α-d-glucose 6-phosphate (G6P) via a bisphosphorylated sugar intermediate, α-d-glucose 1,6-bisphoshate (G16P). G6P is a key molecule in glucose homeostasis, and a substrate of two major metabolic pathways, glycolysis and the pentose phosphate pathway. Conversion of G6P into G1P is an important step in the synthesis of uridine diphosphate glucose, which is used in the cell for glycogen synthesis and as a precursor in protein N-glycosylation^3^.

There are two main *PGM1* isoforms expressed in human cells^11^. Isoform 1 encodes a variant of the PGM1 protein with 562 residues and is ubiquitously expressed in most, if not all tissues^9,11^. The orthologous rabbit (*Oryctolagus cuniculus*) PGM1 protein, displaying 97% sequence identity with the human PGM1 isoform 1, was studied extensively by Ray and co-workers over several decades^12–14^. That work clarified the reversible reaction mechanism of PGM1 and the rabbit PGM1 3D structure^14^. The structure of wild-type isoform 1 of human PGM1 was determined by Beamer and collaborators recently^15^, and the structures of several variants with known pathogenic mutations have also been published^15–17^. Both the rabbit and human PGM1 3D structures have been used to analyze the effect of additional disease-related missense mutations in human PGM1^8,15^.

Isoform 1 encoded PGM1 is a monomeric protein and, as the other members of the α-d-phosphohexomutase superfamily, comprises four domains of roughly the same size organized in what has been described as a “heart shape”^7,15,18^. PGM1 structures of both inactive, unphosphorylated PGM1^15^ and active phosphoenzyme^14,16^ have been reported. Recently, Stiers and Beamer solved the structure of inactive unphosphorylated PGM1-1 in complex with G6P (PGM1-1:G6P)^17^, but until now no pair of structures of both substrate and product complexes for the same activated phospho-enzyme PGM1 variant, crucial for elucidating the detailed reaction mechanism, has been published.

Isoform 2 of human *PGM1* encodes a protein comprising 580 residues, and it differs from isoform 1 by having a longer and different N-terminal^11^. This isoform has not been thoroughly explored, but expression appears to be less widespread than isoform 1. *PGM1* isoform 2 was shown, by Kim and co-workers in 1992, to be expressed at least in skeletal muscular tissue^11,19^. The aim of the current study was to explore the evolution and conservation of the *PGM1* gene and its two isoforms, and determine the 3D structure of human PGM1 isoform 2, including both substrate and product complexes. The results will be useful for interpretation of the pathogenicity of mutations that hit *PGM1* isoform 1, as well as those that exclusively occur in isoform 2. In a recent study to define phenotypic groups in patients with PGM1 deficiency, all but four patients in the cohort demonstrated various congenital malformations^4^. The four patients presented instead with a primary muscle phenotype, and it was suggested that some phenotypic variation due to alterations of isoform 2 of *PGM1* could underlie the primary muscle phenotype in these patients. Apart from this, *PGM1* isoform 2 has been ignored in clinical work, as well as in biochemical investigations, the last 25 years. Here we present three crystal structures of the isoform 2 variant of PGM1, both as a free enzyme and in complex with substrate and product. The structures show the longer N-terminal of this PGM1 variant, and the ligand complex structures reveal for the first time the detailed structural basis for substrate/product recognition by human PGM1.

## Results and Discussion

### Evolution of the PGM1-like protein family

**Figure 1:**
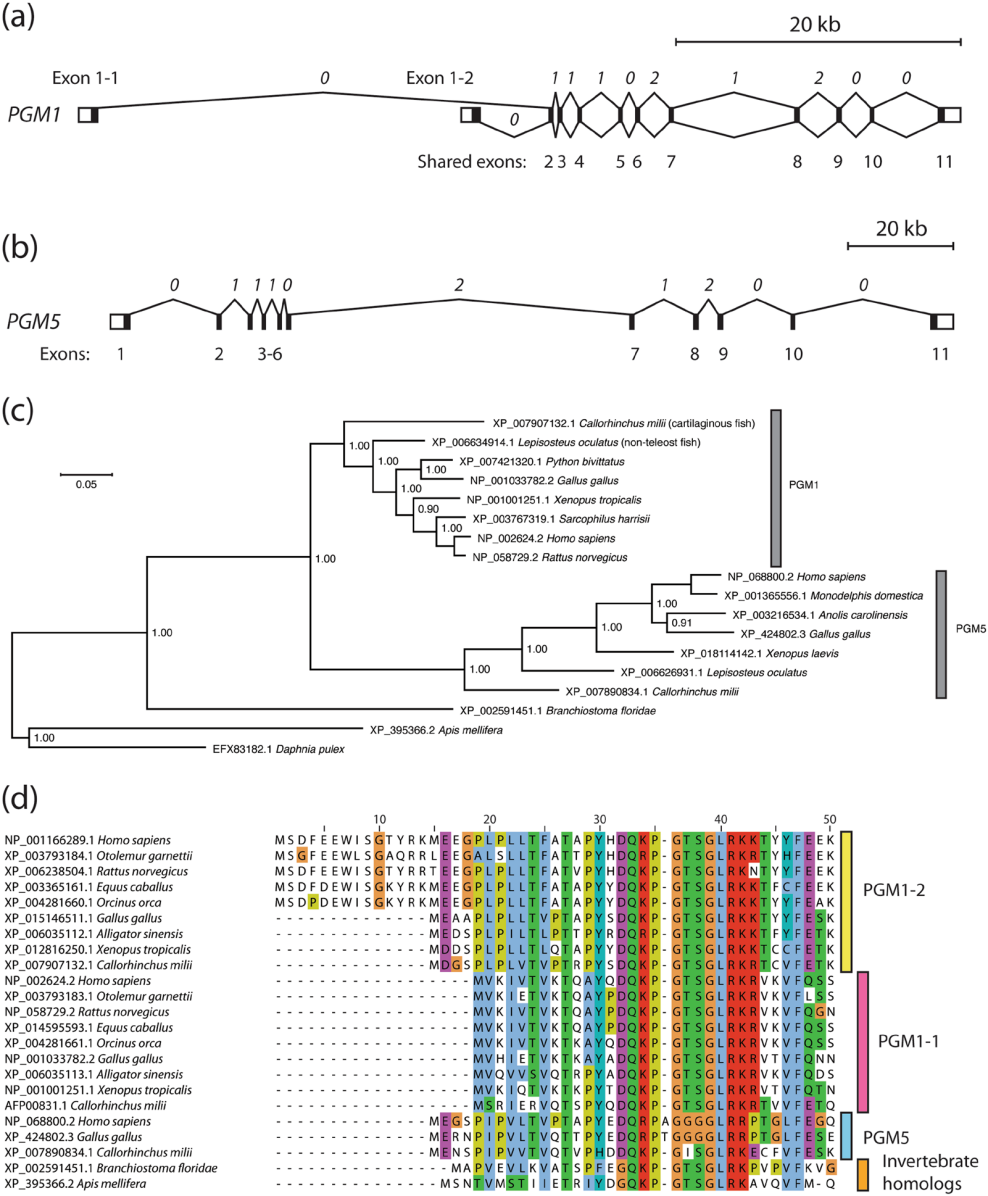
PGM1 sequence evolution. (**a**) Gene structure of human *PGM1* with two alternative transcription start sites in exons 1-1 and 1-2 giving rise to the alternative transcripts translated into PGM1 isoforms 1 (PGM1-1, splicing shown above the exons) and 2 (PGM1-2, splicing shown below exons). 5′ untranslated regions (5′ UTRs) are shown in exons 1-1 and 1-2, and 3’ UTR in exon 11, as white boxes. Intron phases are shown (in italics) above the introns, defined as the position of the intron within a codon, with introns with phases 0, 1, and, 2 located before the first base, after the first base, or after the second base, respectively. The gene is found at chromosome 1p31.3 and has a length of approximately 70,000 base pairs (see also scale bar above gene structure). The exon lengths are not shown at the correct scale. (**b**) Gene structure of human *PGM1* paralog *PGM5* with 5′ and 3′ UTRs shown as white boxes in exons 1 and 11, respectively. Intron phases, conserved and identical to *PGM1*, are shown (in italics) above the introns. The gene is located at chromosome 9p21.11 with a length of approximately 175 kb. (**c**) The *PGM1* and *PGM5* paralogs are the result of a gene duplication event in the common ancestor of the Gnathostomata, of jawed vertebrates. The Bayesian inference tree is based on the alignment of the protein sequences (segment corresponding to exons 2 to 11 in human PGM1, WAG + Γ model with four rate categories). The phylogram is shown with estimated branch lengths proportional to the number of substitutions at each site, as indicated by the scale bar. For each node, Bayesian posterior probabilities are shown. The arthropod clade with the crustacean *D. pulex* and the honey bee (*A. mellifera*) was set as outgroup in order to root the tree. (**d**) Multiple sequence alignment of the N-termini of isoforms PGM1-1 and PGM1-2 from human, northern greater galago, rat, horse, orca, chicken, alligator, a frog and the Australian ghostshark, a cartilaginous fish (binomial names of the species, in the same order, is given in the panel), together with three vertebrate PGM5 sequences and homologs from two invertebrates, the amphioxus and honey bee. Residue numbering for human PGM1-2 is shown above the alignment.

The human *PGM1* gene has two main biologically relevant isoforms resulting from alternative splicing and the use of two alternative promoters and 5′ exons, exons 1-1 and 1-2 in isoforms 1 and 2, respectively, together with shared exons 2–11^11^. In this work, we use the terms PGM1-1 (NCBI RefSeq identifier NP_002624.2) and PGM1-2 (NP_001166289.1) for these two protein variants (Fig. 1a). The protein segments encoded by the two alternative 5′ exons are roughly 51% identical, and the most parsimonious explanation for this gene structure is that exons 1-1 and 1-2 are paralogs, due to an exon duplication event (*vide infra*). Little-studied human *PGM5* is a paralogous gene with the same number of exons and identical intron phases as *PGM1*, but lacking the duplicated 5′ exon (Fig. 1b). Human PGM1 and PGM5 (NP_068800.2) are quite closely related with protein sequence identity at 65%, while other human proteins in this superfamily, PGM2, PGM2L1, and PGM3, are remote homologs with sequence identity to PGM1/5 well below 25%^7,9,20^.

A data set of 146 homologous sequences was collected from public databases, all sequences with sequence identity above 50% compared with human PGM1-1. The sequences were carefully checked, employing publicly available gene browsers and RNA-seq data sets. Only sequences that appeared to represent full-length transcripts with all conserved exons included were used for further analysis. The aligned sequences are shown in Supplementary Fig. S1.

Alignment, comparison and analysis of the sequences, and careful phylogenetic analysis (*vide infra*), showed that 30 of the sequences are PGM5 orthologs, with sequences from placental and marsupial mammals, reptiles, birds, a frog, the coelacanth, the non-teleost spotted gar (*Lepisosteus oculatus*), several teleost fishes, and the Australian ghostshark (*Callorhinchus milii*), a cartilaginous fish (data in Fig. S1). 18 of the sequences are from invertebrates, including the cephalochordate amphioxus (*Branchiostoma floridae*), two tunicates, a hemichordate, two sea anemones, two mollusks, and arthropods such as *Daphnia pulex*, a planktonic crustacean, an arachnid, and the European honey bee (*Apis mellifera*) and turnip sawfly (*Athalia rosae*). Finally, 98 sequences are PGM1 orthologs from all the main vertebrate clades, in most cases both isoforms 1 and 2 (all data in Fig. S1).

A subset of sequences, 7 PGM5 and 8 PGM1 orthologs, chosen to include all main groups of jawed vertebrates, and 3 invertebrate PGM1/5 homologs were aligned, and the segment corresponding to human PGM1 exons 2 to 11 was used to calculate a statistically strongly supported phylogenetic tree employing Bayesian inference methods. The tree was rooted with arthropods as an outgroup (Fig. 1c). Several attempts were also made on determining the phylogenetic relationship of the paralogous exons *PGM1* exon 1-1 (1ex11), *PGM1* exon 1-2 (1ex12), and *PGM5* exon 1 (5ex1). For the human proteins, pairwise sequence identity is 51%, 43%, and 62%, for the pairs 1ex11/1ex12, 1ex11/5ex1, and 1ex12/5ex1, respectively. However, state-of-the-art probabilistic methods, including Bayesian inference methods, were not able to determine, with reasonable statistical support, which pairs of exons, if any, are phylogenetically more closely related to each other than to the third paralog.

Our analysis shows that PGM1 and PGM5 form a clade in animals where many invertebrate species, but not all, have a single PGM1/5 homolog. The Bayesian inference tree in Fig. 1c strongly supports a model where a *PGM1/PGM5* ancestor gene was duplicated in a common ancestor of jawed vertebrates after the branching off of tunicates and cephalochordates, the closest living invertebrate relatives of vertebrates. The 5′ exon of *PGM1* was also duplicated in a common ancestor of jawed vertebrates, the Gnathostomata, but the paralogous exon is not duplicated in extant *PGM5* or the invertebrate homologs. Both the gene duplication leading to *PGM1* and *PGM5* and the exon duplication of *PGM1* exon 1 thus happened more than 420 Myrs ago^21^. Due to the long time since these duplications occurred, and the limited phylogenetic signal in the relatively short paralogous 5′ exons, it is not currently possible to determine if the gene duplicated first, followed by exon 1 duplication limited to *PGM1*, or the alternative hypothesis of an initial exon 1 duplication followed by gene duplication and subsequent loss of exon 1-2 in *PGM5* only. It is also possible that both genome rearrangements took place more or less simultaneously. Clearly, however, our analysis shows that both main isoforms of *PGM1* have roughly the same age as the gene itself.

All main vertebrate clades appear to have kept a single copy of both *PGM1* and *PGM5* and a duplicated *PGM1* exon 1, with the teleost fish exception noted below. Missing sequences in our data set in Fig. S1 is likely due to missing data and not to actual gene- or isoform-loss in these species. Both genes and both *PGM1* isoforms are clearly functionally important in vertebrates as they are conserved since the last common ancestor of cartilaginous fishes and Euteleostomi, the “bony vertebrates”. We found no obvious or wide-spread gene duplications, gene losses, or additional conserved splice variants in *PGM1* or *PGM5* in vertebrates, apart from teleost fishes, where all investigated species, for example the Nile tilapia (*Oreochromis niloticus*) and medaka (*Oryzias latipes*), have a duplicated *PGM1* gene, thus giving two *PGM1* paralogs. Interestingly, it appears that one of the two paralogs have lost exon 1-2, while the second has lost exon 1-1. Teleost fish therefore also have two PGM1 variants, encoded by *two separate genes*, while all other vertebrate groups have the two same variants, isoforms 1 and 2, of the same *PGM1* gene. This is again a strong indication that both *PGM1* isoforms have a crucial and conserved function in all vertebrate species, including the human.

Human PGM1-2 has a longer N-terminus than the well-studied PGM1-1 isoform, with 100 and 82 residues encoded by exons 1-2 and 1-1, respectively. Importantly, the longer N-terminus is conserved in placental mammals, but not in other vertebrate species (Figs. 1d and S1).

### Overall structure of human PGM1 isoform 2

**Figure 2:**
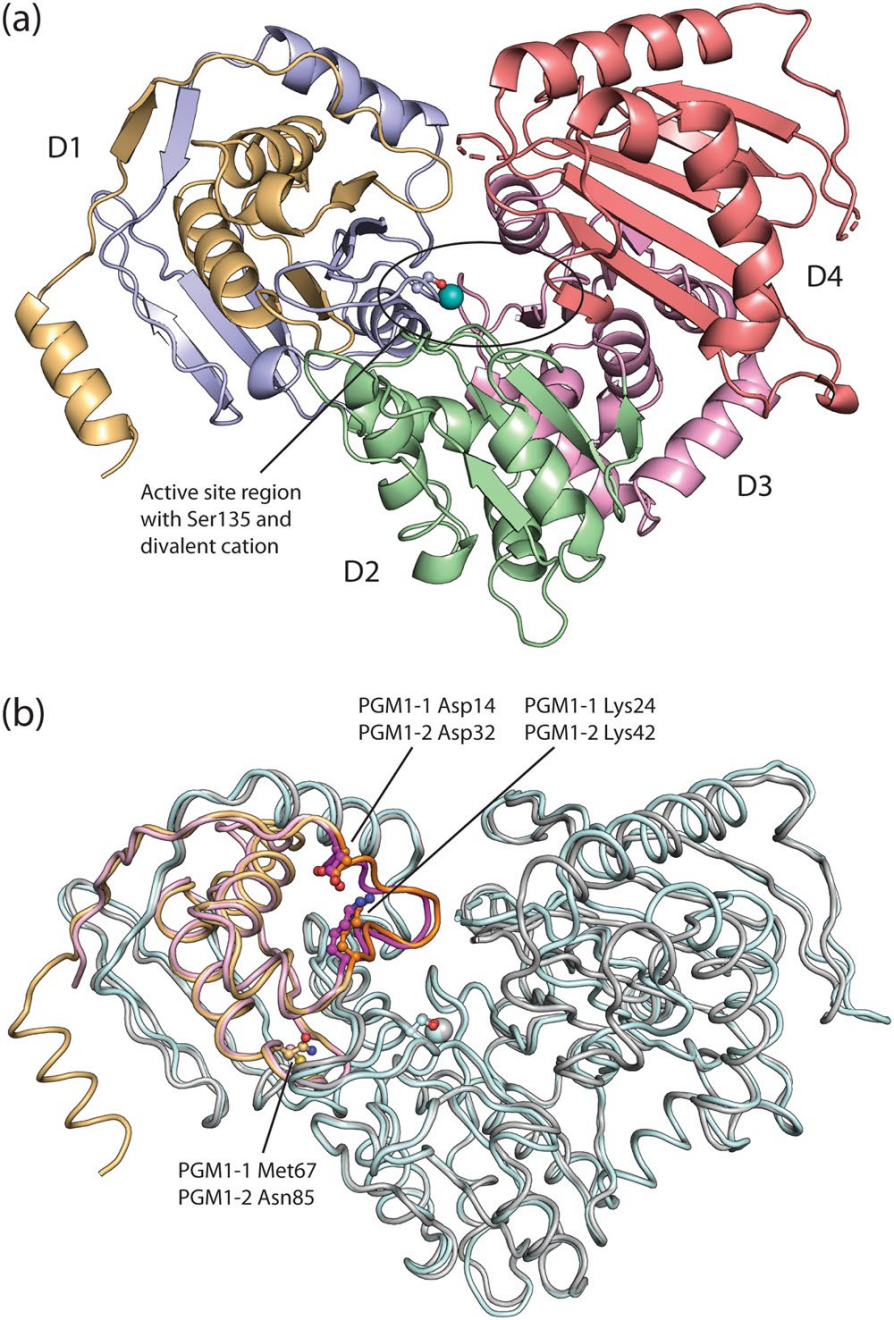
Structure of human PGM1 isoform 2. (**a**) PGM1-2 has four structural domains, D1 to D4, arranged in an overall “heart shape”. Domains D2 to D4 are shown in green (residues 210-322), pink (residues 323-439), and red (residues 440-580), respectively. The segment of D1 encoded by isoform specific exon 1-2 is shown in light orange (N-terminus to residue 100), while the rest of D1 (residues 101 to 209), encoded by exons 2, 3, and partially by exon 4, is colored blue. The active site region, with contributions from all four domains, has a dephosphorylated Ser135 (ball-and-stick representation) and binds a divalent cation (teal sphere). It is located in the central large cleft. (**b**) Structural alignment of the recently solved 3D structure of PGM1-1^15^ (PDB identifier 5EPC, chain A) and PGM1-2 (this work) reveals that the two isoforms are highly similar. The exon 1-1 encoded segment of PGM1-1 D1 is colored pink, while the rest of the protein is shown in pale cyan. PGM1-2 is shown in grey, with the isoform specific exon 1-2 encoded segment in orange. Two isoform specific, paralogous loops, Asp14 to Lys24 in PGM1-1 and Asp32 to Lys42 in PGM1-2, are shown with darker shades of pink and orange, respectively. The loops have identical sequences, very similar structures and are the only parts of the exon1-1/exon1-2 encoded segments that are relatively close to the active site. PGM1-1 Met67 and PGM1-2 Asn85 is the pair of paralogous non-identical residues that are closest to the active site (>10 Å), strongly suggesting that PGM1-1 and PGM1-2 have identical active sites. The figure is rendered as a Cα trace.

The crystal structure of the isoform 2 variant of human PGM1 was determined, and is reported here for the first time. The structure was solved both for the free holoenzyme form and in complex with substrate (G1P) and product (G6P). In all three cases, the space group was identical (*P*2_1_), with a single PGM1-2 protein chain in the asymmetric unit. Final statistics for all three data sets are shown in Table 1. Secondary structure elements are shown in Supplementary Fig. S2 and listed in Supplementary Table S1. Apart from the extended N-terminal, the overall PGM1-2 2.8 Å enzyme structure (Fig. 2a) is very similar to the recently determined PGM1-1 structure (Fig. 2b)^15^. The PGM1-2 monomer with overall dimensions 45 × 65 × 80 Å adopts the evolutionary conserved “heart shape” comprising four roughly equal sized domains 1 to 4, D1 to D4 (Fig. 2a). As previously described in the reports on the structures of rabbit PGM1 and human PGM1-1^14,15^, D1 to D3 share a common mixed α/β core, with a central four-stranded β-sheet sandwiched between two α-helices. D4 contains a six-stranded antiparallel β-sheet with two α-helices lying on the face of the sheet opposite the active site. The active site is located centrally, in a deep cleft between the four domains (Fig. 2a) and is mainly formed by one conserved loop structure contributed by each of the four domains (See also Fig. S1). The first loop is the active site Ser-containing loop in D1. Enzymatically active human PGM1 contains a phosphoryl group (PO_3_^2-^) esterified with the γ-hydroxyl group of a conserved phosphoserine residue, p-Ser117 in PGM1-1 and p-Ser135 in PGM1-2. In the following, residue numbering according to the PGM1-2 isoform will be used. The three remaining active site loops are the metal-binding loop in D2, interacting with the divalent cation required for enzymatic activity, the sugar-binding loop in D3, and finally, the phosphate-binding loop in D4. This last loop interacts with the substrate phosphate moiety, not the phosphate group on p-Ser135. Heterologously expressed PGMs are usually found to have a mixture of unphosphorylated and phosphorylated active site phosphoserine residues^22^. Nevertheless, Ser135 is found in the dephosphorylated state in the free holoprotein crystal structure, as the corresponding active site residue was in the recently published PGM1-1 structure^15^.

**Table 1:** Data collection and refinement statistics.

	PGM1-2	PGM1-2:G1P	PGM1-2:G6P
**Data collection**			
Beamline	ESRF, ID29	ESRF, ID29	ESRF, ID29
Wavelength (Å)	0.9762	0.9762	0.9762
Temperature (K)	100	100	100
Space group	*P*2_1_	*P*2_1_	*P*2_1_
Cell dimensions			
*a*, *b*, *c* (Å)	72.34, 53.38, 76.07	72.95, 53.35, 76.32	73.26, 53.16, 76.83
β	100.15°	98.44°	99.14°
Resolution (Å)	43.47-2.75 (2.90-2.75)^a^	43.57–2.70 (2.83–2.70)	48.63–2.70 (2.83–2.70)
*R*_merge_	0.174 (0.797)	0.109 (0.781)	0.082 (0.560)
Mean *I*/σ*I*	6.9 (2.0)	10.6 (1.9)	13.1 (2.1)
Completeness (%)	99.7 (99.9)	99.6 (99.6)	99.6 (99.7)
Redundancy	5.0 (5.1)	4.9 (4.9)	4.6 (4.8)
**Refinement statistics**			
Resolution (Å)	43.47–2.75	43.57–2.70	48.63–2.70
No. of reflections	15,063 (1482)	16,141 (1605)	16,195 (1600)
*R*_work_/*R*_free_	0.209/0.270	0.197/0.248	0.206/0.246
No. of atoms			
Protein	4411	4440	4401
Ligand/ion	1	17	17
Water	57	49	84
*B*-factors (Å^2^)			
Protein	52.44	56.06	47.07
Ligand/ion	38.70	57.33	63.62
Water	43.89	46.77	40.32
RMS deviations^b^			
Bond lengths (Å)	0.003	0.003	0.002
Bond angles (°)	0.52	0.56	0.49
Ramachandran statistics^c^			
Favored (%)	97	96	96
Allowed (%)	3	4	4
PDB code	6SNP	6SNO	6SNQ

^a^Values in parentheses are for highest-resolution shell.

^b^Root mean square deviations.

^c^Ramachandran plots generated with MolProbity via the PDB validation server.

The structural alignment of the PGM1-1 and PGM1-2 structures gives a full-length Cα root mean square deviation (RMSD) of 1.4 Å (Fig. 2b), but a structural superposition of exons D1-D3 gives a Cα RMSD of only 0.64 Å for this mainly rigid part of the protein. Given this alignment, D4 is found to be rotated roughly 10° in the current PGM1-2 structure compared with the previously published PGM1-1 structure^15^. The rotation of D4 relative to the rest of the protein appears to be important for substrate binding and product release, but also for reorientation of the bisphosphorylated reaction intermediate^17^. In their report on the crystal structures of the related protein phosphomannomutase/phosphoglucomutase (PMM/PGM) from *P. aeruginosa* in complex with biological ligands, Regni *et al*.^23^ describe a rotation of D4 relative to the rest of the protein by approximately 9°, resulting in the movement of individual residues up to 4.5 Å. Comparing the human PGM1-1 and PGM1-2 structures, some D4 residues indeed move more than 4 Å, but the residues in the phosphate-binding D4 loop are displaced approximately 2 Å in PGM1-2 giving a slightly compressed active site cleft compared with the PGM1-1 structure.

Exon 1-1 encodes the 82 N-terminal of the 191 D1 residues of PGM1-1, and an alignment of this segment with the 82 paralogous residues of PGM1-2 shows a sequence identity of 51% (42 of 82 residues, no insertions or deletions). Structural superposition of these 82 residues of the PGM1-2 enzyme structure with the same segment of the PGM1-1^15^ reveals that the structures of the exon 1-1 and 1-2 encoded segments of D1 are highly similar with an RMSD of only 0.87 Å for 82 Cα pairs (alignment as shown in Fig. 2b). Clearly, the two isoforms of PGM1 are structurally overall very similar and the full four-loop active site, including the Ser-containing loop contributed by D1, is encoded by identical protein sequences. There is more than 14 Å between the divalent cation of the active site and the spatially nearest residue pair that is different in PGM1-1 (Met67) and PGM1-2 (Asn85) (Fig. 2b). The only loop in D1 encoded by alternative exons 1-1 and 1-2 that is pointing towards the active site is formed by residues 14 to 24 in PGM1-1 and 32 to 42 of PGM1-2 (Fig. 2b). These two paralogous loops are separated by an evolutionary distance of more than 420 Myrs (*vide supra*), but the sequences are nevertheless identical and with very similar conformations in PGM1-1 and PGM1-2 (Fig. 2b). The strong conservation of this fifth loop (See also Fig. S1) suggests that it too is important for PGM1 enzymatic activity, even though it is not one of the four previously discussed active site loops of the PGMs and other enzymes of the α-d-phosphohexomutase superfamily^7^. In summary, the active sites of the PGM1-1 and PGM1-2 variants appears to be close to identical.

### Comparison between PGM1 free enzyme and complex structures

**Figure 3:**
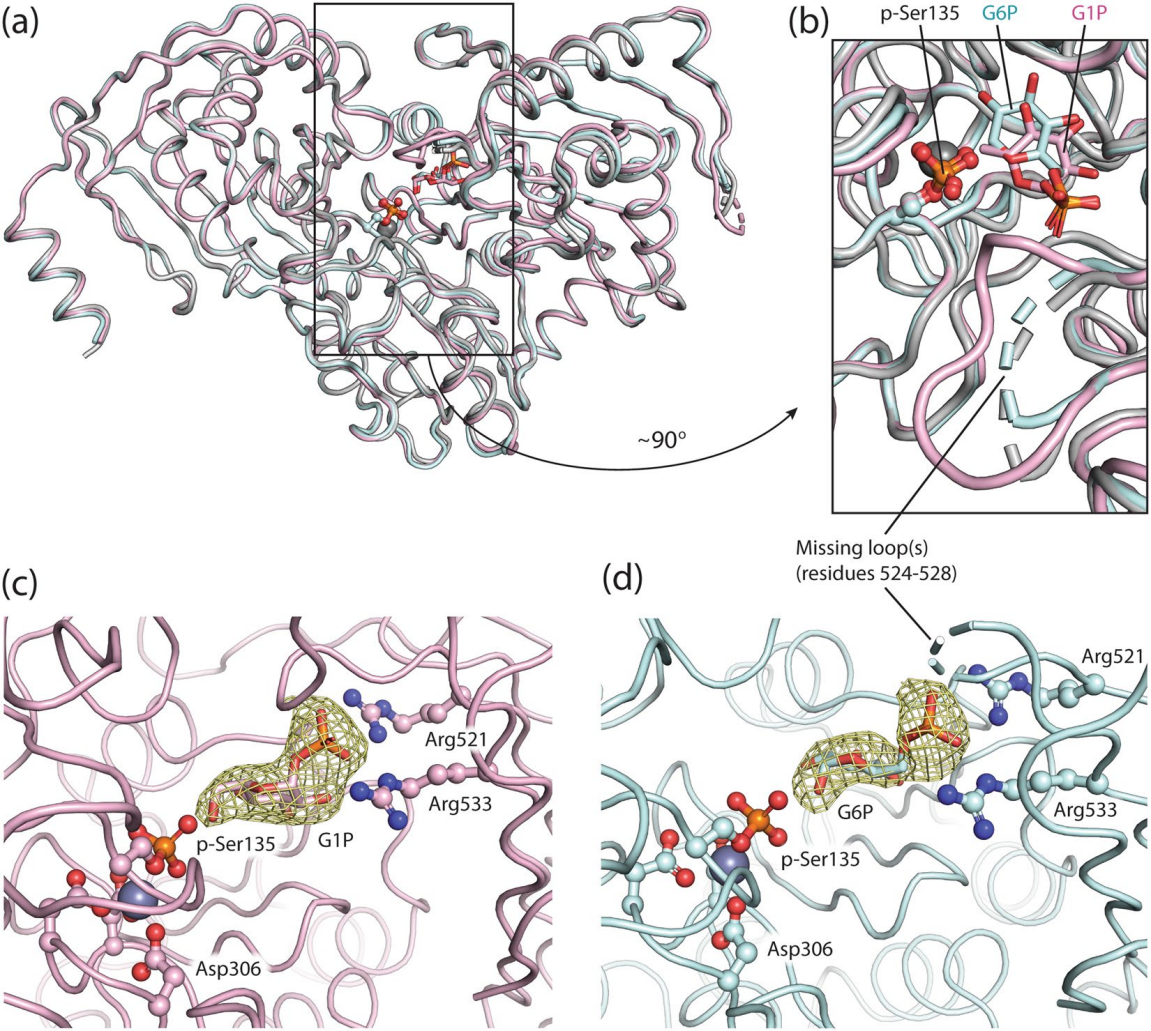
Comparison of free PGM1-2 and substrate and product complexes. (**a**) Structural alignment of PGM1-2 with no bound ligand (grey) and the complexes PGM1-2:G1P (pale pink) and PGM1-2:G6P (light cyan), rendered as a Cα trace, shows that ligand binding does not affect overall structure. Ligands are shown in sticks representation, phosphoserine (p-Ser135) as ball-and-sticks, and the active site divalent cation as a sphere (grey). (**b**) Active site region magnified, with coloring and rendering as in panel (**a**) (rotated roughly 90° out of the plane). (**c**) Polder OMIT map for G1P in the PGM1-2:G1P complex contoured at 4.0σ strongly supports the presence of the ligand (sticks rendering) in the crystal structure. Selected active site residues, p-Ser135, phosphate-binding Arg521 and Arg533, as well as Asp306, Asp308, and Asp310 complexing the divalent cation (grey sphere), are shown as ball-and-sticks. (**d**) Polder OMIT map for G6P in the PGM1-2:G6P complex contoured at 3.5σ.

PGM1-2 substrate and product complexes were generated by soaking crystals with G1P and G6P, respectively. We here publish, for the first time, data for a eukaryotic PGM that show the detailed structures of both the pre- and post-reaction complexes. The overall structures of the two PGM1-2 complexes are very similar to each other and to the holoprotein structure with no bound ligand. The superposition of the three structures (PGM1-2 free protein, PGM1-2 in complex with G1P, and PGM1-2 in complex with G6P) gave a Cα RMSD of only 0.67 Å or less (Fig. 3a,b). Only minor differences can be seen when comparing the free protein structure with the complex structures, with D4 rotated 2° or less in the complexes, compared to the free protein structure. Similarly, in a recent study of complex structures and free protein for a bacterial phosphoglucomutase^24^ conformational variability was not observed for D4. It was speculated that this might be due to tight packing in the crystal lattice. It cannot be excluded that longer soaking times, or crystallization of preformed complexes, might have given larger rotations for D4 in the complex structures compared to the free enzyme.

The PGM1-2 free protein and complex structures all contain a divalent cation, complexed by the metal-binding loop in D2 and Ser135 of D1 (Figs. 2 and 3), similar to earlier published PGM1 structures^14–17^. Unlike the PGM1-2 (Fig. 2) and PGM1-1 (Fig. 2b)^15^ free holoprotein structures, the active site Ser135 residue in D1 is phosphorylated in the PGM1-2 substrate and product complexes (Fig. 3). While no attempt was made on preparing PGM1-2 that was fully phosphorylated at p-Ser135, it has also previously been found that treatment of PGM homologs with high concentrations of monophosphosugars, as in the current crystallization setup, can lead to a shift in enzyme activation state towards a higher degree of phosphorylation^22,25^. Finally, the complex structures have strong densities for G1P and G6P in their active sites (Fig. 3c,d).

### Structural and functional differences between the two isoforms of human PGM1

The additional residues at the PGM1-2 N-terminus form a short α-helix that protrudes from D1 in the crystal structure (Fig. 2b). There is poor packing between the N-terminal α-helix and the rest of PGM1-2 D1 (Supplementary Fig. S3), and this segment is predicted to be structurally disordered by prediction software (Supplementary Fig. S4). The longer PGM1-2 N-terminus is only found in placentals, while PGM1-2 in other vertebrates, PGM1-1 and PGM5 in all vertebrates, and the invertebrate PGM homologs all have roughly the same length of the N-terminus (Figs. 1d and S1). The placental PGM1-2 N-terminal extension is enriched in charged and aromatic residues (Supplementary Fig. S5a,b), but the only residues that are fully or nearly completely conserved in all placentals investigated are Trp7 and Ile8 (Fig. S5a). The hydrophobic side chains of these residues are, surprisingly, solvent exposed in the crystal structure. They are not interacting with other protein chains in the crystal. All in all, the orientation and interactions of the α-helical human PGM1-2 N-terminal extension might be due to crystal packing, and the protein N-terminal tail may be more flexible or partially unstructured for the free protein in solution. The most parsimonious explanation for the lack of long N-terminus in non-placental PGM1-2 is that this extension is a relatively new feature of PGM1-2 that evolved in a common ancestor of placentals. The long N-terminus is clearly not necessary for general PGM1-2 function in most vertebrates, as it was not in the ancestors of placentals.

The average half-life of gene duplicates in eukaryotes is only 4 Myrs^26^. The only duplicated genes that survive to a state where both copies are maintained by natural selection, are those where both genes have evolved separate biologically important functions. Similarly, duplicated transcript and protein variants due to exon duplications, as for PGM1-1 and PGM1-2, will not survive more than a few million years unless the variants both have obtained separate important functions in the organism. PGM1-1 and PGM1-2 have identical active sites (*vide supra*) and otherwise very similar structure, and the most obvious difference between two isoforms, the extended N-terminus, is only found in a subgroup of mammals, the placentals. It is thus not immediately clear what the biological function of PGM1-2 is, and how it differs from widely studied PGM1-1.

Expression levels from several hundred human subjects, available from the Genotype-Tissue Expression (GTEx) project^27^, show the highest *PGM1* transcript levels in skeletal muscle tissue. PGM1-1 appears to be highly expressed in skeletal muscle, and at low to intermediate levels in all other investigated tissues, while PGM1-2 is only found at significant levels in skeletal muscle in the current GTEx data. However, even in skeletal muscle tissue the PGM1-1 transcript levels are one to two orders of magnitude higher than PGM1-2. It appears that PGM1-2 has some specialized and evolutionary conserved function in skeletal muscle, but also in these tissues it seems likely that PGM1-1 provides the main phosphoglucomutase activity.

Sequence logos were generated for PGM1-1 and PGM1-2 from multiple sequence alignments of 42 and 43 vertebrate homologs, respectively. The logos for the segments encoded by exons 1-1 and 1-2 are aligned and compared in Fig. S5b,c. Some parts of the 5′ exon-encoded protein segment is more or less fully conserved in both PGM1-1 and PGM1-2, for example the 5^th^ active site loop discussed above (Fig. S5b). Other examples are PGM1-2 Tyr30 and Tyr53 which are involved in structurally important stacking interactions in D1, and the motif between PGM1-2 residues 76 and 82 that builds the core of D1.

**Figure 4:**
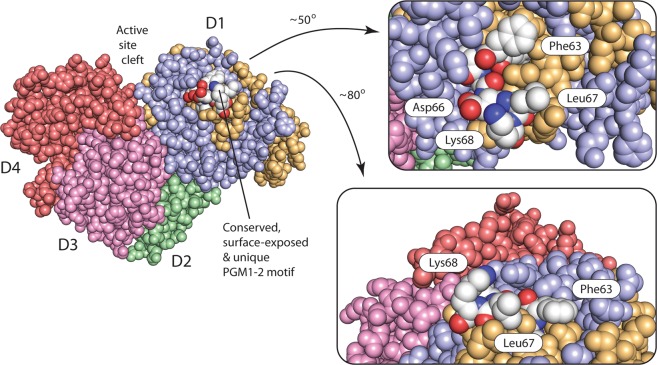
PGM1-2 has a unique, surface-exposed, highly conserved patch of residues. A conserved FFSIDLK motif (residues 62 to 68, with carbons, nitrogens, and oxygens, as white, dark blue, and dark red, respectively) is unique to PGM1-2. Surface exposed Phe63, Asp66, Leu67, and Lys68 (only conserved as positively charged) are completely conserved in vertebrate PGM1-2 and are ideally located to interact with a PGM1-2 specific binding partner. The PGM1-2 protein is shown as a space-filling CPK calotte model with identical coloring as in Fig. 2a. Domains D4, D3, and D2 are shown in red, pink, and green, respectively. The PGM1-2 isoform specific exon 1-2 encoded segment is shown in light orange/white, while the rest of D1 is colored blue. Parts of the PGM1-2 structure (left) are shown magnified and rotated roughly 50° (top, right) and 80° (bottom, right) around two different axes.

Some few residues are conserved in one PGM1 isoform, but are unconserved or conserved as something else in the other isoform, possibly pointing to unique isoform-specific function (Fig. S5b,c). The only evolutionary conserved segment found in PGM1-2 and not in PGM1-1, which also is exposed on the surface of the protein, is the FFSIDLK motif between residues 62 and 68 (Fig. S5c). The residues of this PGM1-2 specific motif are highlighted in the free enzyme PGM1-2 structure in Fig. 4. Lys68 is conserved as positively charged Lys/Arg in all vertebrates, including cartilaginous fish, while Asp66, Leu67, and Phe63 are absolutely conserved in vertebrates, the two latter even with hydrophobic side chains exposed to the solvent. It is tempting to speculate that this evolutionary conserved patch of residues is involved in a specific and highly conserved interaction between PGM1-2 and a macromolecular binding partner, and that this interaction is unique to PGM1-2, and not present for PGM1-1. It is possible that this PGM1-2-specific interaction is also complemented by interactions mediated by the N-terminal extension in placental PGM1-2. There are, interestingly, no conserved segments/patches only found in PGM1-1 that suggests that this isoform has a unique binding partner.

There are no indications that mammalian PGM1-1 is not functioning as a monomer in solution^7,28^, but some bacterial homologs have been shown to be forming dimers in solution^28^, in some cases mediated by an N-terminal extension on D1^29^. From visual inspection of our current crystal structures, there are no indications that the PGM1-2 N-terminal extension is involved in homo-multimerization in solution. Predictions from the macromolecular assembly predictor PISA^30^ strongly suggest that human PGM1-2, as PGM1-1, is not forming a multimer with itself in solution.

### Recognition of G1P substrate and G6P product in the PGM1 active site pocket

Structural superposition of the PGM1-2:G1P and PGM1-2:G6P complexes shows that the active site loop residues involved in the recognition of the two ligands are, to a large degree, the same, and in similar conformations. The interactions involving each of the four active site loops are discussed in detail below.

**Figure 5:**
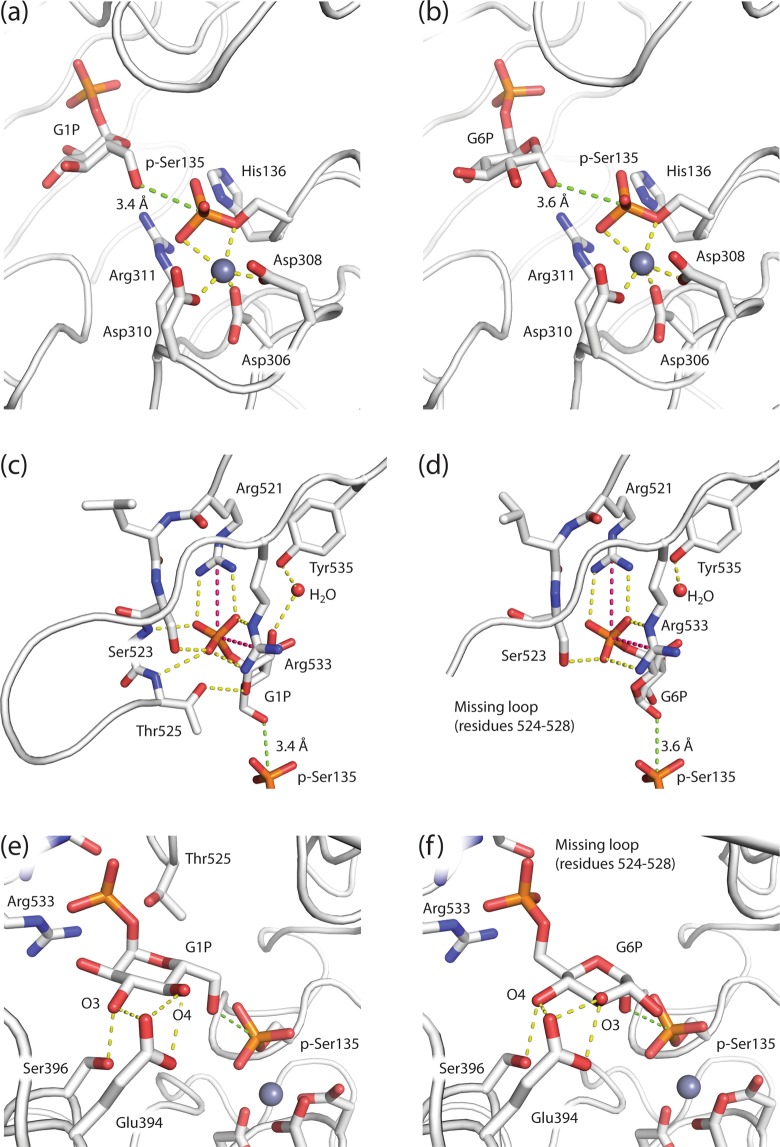
Comparison of the PGM1 substrate and product complex active sites. (**a**) Substrate (G1P) and (**b**) product (G6P) are located with O6 and O1, respectively, roughly 3.5 Å (green dotted line) from the P atom of the phosphoserine (p-Ser135) of the PGM1 active site. The divalent cation (grey sphere) is complexed by three Asp residues of the metal-binding loop in D2 and the p-Ser group (yellow dotted lines). Both substrate (**c**) and product (**d**) are being anchored in the active site by strong, bi-dentate interactions (pink and yellow dotted lines) to Arg521 and Arg533 in the phosphate-binding loop of D4. Due to lacking electron density, residues 524 to 528 are not visible in the PGM1-2:G6P structure. With the hexose phosphate group anchored by the phosphate-binding loop, the residues Glu394 and Ser396 of the sugar-binding loop in D3 recognizes the hydroxyls in their equatorial positions at C3 and C4 in substrate G1P (**e**). The same two residues, in essentially identical conformations, contact the two hydroxyls, but in swapped positions, in the product complex (**f**).

The active site Ser-containing D1 loop contains the catalytically activated, phosphorylated, p-Ser135 in the PGM1-2:G1P (Fig. 5a) and PGM1-2:G6P (Fig. 5b) complexes. The divalent cations in the active sites are in both structures complexed by three conserved Asp residues (Asp306, Asp308, and Asp310) contributed by the D2 metal-binding loop. The metal ions in the two complex crystal structures are most likely Zn^2+^, due to the crystallization and soaking conditions (see Experimental procedures), rather than Mg^2+^ that is required for enzymatic activity, explaining the stability of substrate and product complexes for structural studies. The divalent cation is also complexed by the phosphoryl group of p-Ser135, which again appears to be interacting with the side chains of His136 and Arg311.

**Figure 6:**
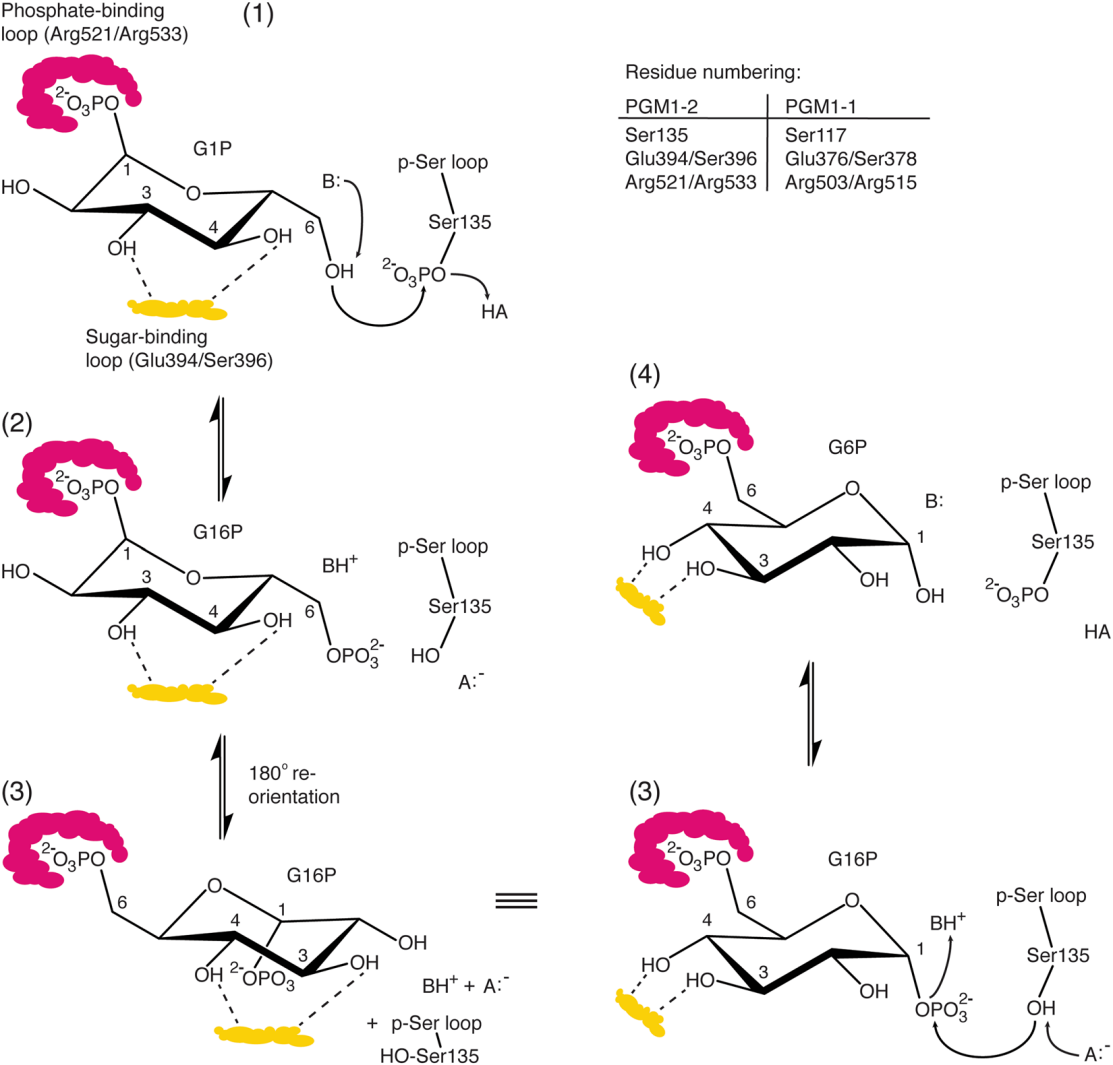
Detailed PGM1 catalytic mechanism for the reversible conversion of G1P to G6P. In the substrate complex (1), the phosphate-binding loop (red) is anchoring the G1P phosphoryl group, while equatorially located hydroxyls at C3 and C4 are recognized by the sugar-binding loop (yellow). The hydroxyl group at C6 is ideally located for attack on the p-Ser phosphoryl group. After phosphoryl-transfer (2) the bisphosphorylated glucose (G16P) is poised for a 180° reorientation (“flip”) in the active site, most likely accompanied with an opening of the active site due to the flexibility of D4. Upon reorientation, G16P is again interacting with the sugar-binding loop through C3 and C4 hydroxyls, but these groups are now interchanged (3). Finally, the phosphoryl group is transferred back to the active site Ser residue on D1 (4), reactivating the PGM1 enzyme.

In the PGM1-2:G1P substrate complex (Fig. 5a), the hydroxyl group of G1P C6 is located 3.4 Å from the p-Ser135 phosphoryl group and ideally located for a nucleophilic substitution attack and transfer of the phosphoryl group, for formation of the bisphosphorylated G16P intermediate. The reaction mechanism is illustrated in Fig. 6. Similarly, the PGM1-2:G6P product complex (Fig. 5b), that is, the substrate complex for the reverse reaction (Fig. 6), has the hydroxyl group of G6P C1 located 3.6 Å from the p-Ser135 phosphoryl group. Overall, the conformations of the active sites around p-Ser135 in the forward and reverse reaction substrate complexes (Fig. 5a,b) are strikingly similar. In both reactions, the divalent cation is likely to function as an electron withdrawing group, facilitating the transfer of the phosphoryl group to the glucose and the breaking of the p-Ser135 P-O bond. It is tempting to speculate that also conserved residues His136 and Arg311 are taking part in the phosphoryl transfer reaction, either as electron withdrawing groups (Arg311) or proton accepting base (His136) (Fig. 6). A third active site residue, Lys407 in human PGM1-2, have previously been suggested to be involved in the acid-base catalysis in the PGM family of proteins^31^. However, in our complex structures, the ε-amino group of Lys407 is located 9.4 Å from the G1P C6 hydroxyl group, and 9.1 Å from G6P C1 hydroxyl, in PGM1-2:G1P and PGM1-2:G6P, respectively. Arg311 and His136 are within 4 Å and 5.5 Å of the same hydroxyls in both complexes. Most likely, all three residues, His136, Arg311, and Lys407, together with solvent molecules that are not visible in the structures, are involved in the acid-base chemistry required for catalysis. The LTASHNP sequence of the active site p-Ser-containing D1 loop (Ser135 underlined) and the FDGDGDR sequence of the metal-binding D2 loop (metal complexing residues Asp306, Asp308, and Asp310 underlined) are close to 100% conserved in PGM1 in vertebrates and in the orthologous PGMs of invertebrates (Fig. S1), reflecting the importance of these active site loops for catalysis.

The phosphate binding D4 loop, comprising residues 521 to 534, is located at the opposite side of the active site with respect to p-Ser135. It interacts with the phosphoryl group of the substrate G1P (Fig. 5c), product G6P (Fig. 5d), or intermediate G16P and anchors the ligand via this group through several strong interactions (Fig. 6). The main residues involved in these interactions are Arg521 and Arg533, at each end of the loop, both forming strong, bidentate H-bonds to the phosphate group, as well as Ser523, also H-bonding to the phosphate moiety (Fig. 5c,d). It has previously been noted^15,17^, that poor electron density within this region suggests structural flexibility in the D4 loop. In particular, this can be seen in structures without ligand, indicating that the phosphorylated sugar ligand stabilizes the phosphate binding loop^17^. In our structures, the phosphate binding loop partially displays poor electron density for all three structures, but in the case of PGM1-2:G1P it was possible to model the whole loop. No electron density for residues 524 to 528 was observable for PGM1-2:G6P and PGM1-2 without bound ligand, and these are missing in the structures (Figs. 3b,d and 5d). In the PGM1-2:G1P complex, the backbone amide groups at residues 524 and 525 are interacting with the sugar phosphate group, and also the side chain of Thr525 is seen to H-bond to the glucose, in this case to O5 of the G1P ring (Fig. 5c). The main phosphate interacting residues, Arg521, Ser523, and Arg533 are highly conserved in vertebrate PGM1 (Fig. S1), and are interacting with the sugar phosphate group in nearly identical conformations in the substrate (Fig. 5c) and product (Fig. 5d) complexes. The structural flexibility of the phosphate binding loop in D4 is very likely necessary for efficient “flipping” (Fig. 6) of the bisphosphorylated G16P intermediate, together with the non-rigidity of the interaction between D4 and the three remaining domains.

The sugar-binding loop of D3 is involved in an H-bonding network with the C3 and C4 hydroxyl groups of G1P (Fig. 5e) and G6P (Fig. 5f) which is nearly identical in substrate and product complexes, but with C3 and C4 hydroxyls in swapped positions (Fig. 6). These interactions are crucially important for PGM1 catalytic activity, and this is reflected in the complete conservation of the GEESFGT sequence (Glu394 and Ser396 underlined) of the sugar-binding loop in all metazoan PGM1 homologs (Fig. S1).

### The complex structures elucidate PGM1 substrate specificity

The α-d-phosphohexomutase (PHM) superfamily is traditionally divided into four major subgroups, the PGMs with specificity for α-d-glucose 1- or 6-phosphate, the PMM/PGMs with dual specificity for α-d-glucose and α-d-mannose phosphosugars, and the phosphoglucosamine mutases and *N*-acetylglycosamine phosphomutases. The latter two have specificity for α-d-glucose 1- or 6-phosphate with the hydroxyl group at C2 substituted with an amine group or a larger amide group, respectively^7^. In its lowest energy chair-type conformation, α-d-glucose has the anomeric carbon (C1) substituent in the axial position, but all remaining ring substituents in equatorial positions (Fig. 6). α-d-mannose, the second substrate for the PMM/PGMs, has an identical structure, except for an axial position for hydroxyl on C2, thus all α-d-phosphohexomutases are specific for a particular type of phosphorylated α-d-hexose, and the substrates only differ in the substituent on the C2 carbon of the sugar ring.

The substrate specificity is to a large degree explained by our PGM1 complex structures. With α-d-hexose 1- or 6-phosphates anchored in the active site by their phosphoryl groups, only hexoses with equatorially located hydroxyl groups at both positions C3 and C4 will be able to form strong H-bonding interactions with the highly conserved sugar-binding loop (Fig. 5e,f). This excludes for example d-galactose and d-altrose with C3 and C4 hydroxyls on the same side of the hexose ring. Efficient catalysis for β-anomers are prevented since the 1-phosphates have equatorially located C1 substituent, which very likely is incompatible with sugar-binding loops interactions at C3 and C4 hydroxyls. In addition, C1 hydroxyl in the β-anomer will not be ideally positioned for phosphoryl transfer from p-Ser in the 6-phosphates. The only part of the hexose substrate that is not tightly and specifically interacting with either the phosphate-binding, sugar-binding, or p-Ser containing loops is the C2 carbon and its substituent, explaining that only at this position is there variation in substrate specificity in the PHM superfamily. The Beamer group recently published 15 crystal structures of a PMM/PGM from the γ-proteobacterium *Xanthomonas citri*, including complexes with 1- and 6-phosphohexoses and the G16P intermediate^24^. This work confirms that the loops and residues involved in PHM catalysis are conserved from bacteria to human. For example, the residues directly contacting hexose C3 and C4 hydroxyls (PGM1-2 Glu394 and Ser396) and the residues anchoring the hexose phosphate group in D4 (PGM1-2 Arg521, Ser525, and Arg533) are all conserved and in identical or very similar conformations in the human PGM1-2 and *X. citri* PMM/PGM G1P and G6P complexes. However, compared with our complex structures, the *X. citri* PMM/PGM:G1P and PMM/PGM:G6P structures have a much more open active site, with the G1P and G6P hydroxyl groups involved in nucleophilic attack located more than 6.5 Å from the P atom of the phosphoserine, compared with approximately 3.5 Å in our structures (Supplementary Fig. S6). The *X. citri* PMM/PGM G1P and G6P complex structures therefore appear to correspond to energy minima significantly earlier and later, respectively, along the reaction coordinate, compared with our PGM1-2:G1P and PGM1-2:G6P.

It is less clear from available structures (present results and in Stiers *et al*.^24^ and references cited therein) why the PMM/PGMs have roughly the same activity on both α-d-mannose and α-d-glucose 1-phosphate substrates, while the PGMs have an activity that is several orders of magnitude higher for the latter^7,32^. Firstly, in the *X. citri* PMM/PGM:G1P complex there is an Arg residue interacting with hydroxyls both at C2 and C3^24^, but this residue is not conserved in metazoan PGMs. It is therefore not a universal axial C2-hydroxyl “reader”, and in our human PGM1-2:G1P complex there are no residues interacting directly with the C2 hydroxyl. Secondly, in the G6P complexes of both *X. citri* PMM/PGM^24^ and human PGM1-2, there are no direct interactions between the protein and the C2 hydroxyl group. Finally, there are no moieties in PGM1-2 in our complex structures in a position that appears to be able to block an axial C2 hydroxyl on the hexose, as found in α-d-mannose phosphate. It is possible that the preference for α-d-glucose over α-d-mannose phosphates for PGM1 and the PGM family enzymes is connected with unique processive properties of the PHM enzymes. G16P intermediate has been shown to go through a number of reorientations and catalytic cycles before product is released in PGM enzymes^7,33^. Future work, including sophisticated kinetics studies, is likely to elucidate this.

## Methods

### Materials

All chemicals were of reagent grade and purchased from Sigma-Aldrich unless otherwise noted.

### Expression and purification

The coding region of full length human PGM1 isoform 2 was synthesized with codon usage optimized for expression in *Escherichia coli* (Genscript) and subcloned into the NcoI and BamH1 sites of the pETM-11 vector (EMBL collection) to give an N-terminal hexahistidine tag and a tobacco etch virus (TEV) protease cleavage site fused to the protein. The expression vector was then transformed into the *E. coli* expression strain BL21-CodonPlus (DE3)-RIL for recombinant expression (Agilent Technologies). The cells were grown in LB medium supplemented with 50 μg/ml kanamycin at 37 °C until an OD600 of ~0.7 and the expression was induced by addition of isopropyl-β-d-thiogalactopyranoside to a final concentration of 0.25 mM. Induced cells were grown for 18 hours at 18 °C prior to harvesting by centrifugation at 6000 g for 30 min. Cell pellets were resuspended in lysis buffer (50 mM Tris-HCl pH 8.0 and 300 mM NaCl) and lysed by sonication. A cell free protein extract was prepared by centrifugation, and applied to Ni-NTA resin equilibrated in lysis buffer for approximately 1 hour. The lysate-Ni-NTA mixture was extensively washed with lysis buffer and bound protein was released from the resin using lysis buffer with 300 mM imidazole. After dialysis into 20 mM Tris-HCl pH 8.0, 100 mM NaCl and 10 mM β-mercaptoethanol, and cleavage with His-tagged TEV enzyme, the protein was again applied to equilibrated Ni-NTA resin. The hexahistidine tag and the TEV protease were retained in the Ni-NTA whereas the cleaved protein eluted in the flow through. The purified protein was concentrated to about 12 mg/ml using Amicon Ultra Centrifugal Filters. The yield of purified PGM1 isoform 2 from one liter of *E. coli* was approximately 2 mg.

### Crystallization and data collection

PGM1 isoform 1 was crystallized using the hanging drop vapor diffusion method. For crystallization of the protein with no ligand present, a 1.0 μl protein droplet was mixed with 1.0 μl of precipitant solution containing 0.2 M NH_4_Cl, 0.01 M MgCl_2_, 0.05 M HEPES sodium salt pH 7.0, 2.5 M 1,6-hexanediol, and equilibrated against the precipitant reservoir at room temperature. The same procedure was used for the complex structures, but with a precipitant solution containing 0.10-0.15 M KCl, 0.01 M MgCl_2_, 0.05 M Na cacodylate pH 6.5, 12.5-15% w/v PEG4000. Crystals of PGM1 isoform 2 grew within a few days. Before flash freezing in liquid nitrogen, the crystals were briefly soaked in mother liquor added 30% (v/v) glycerol. Complexes were made by soaking crystals in cryo solution containing either α-d-glucose 1-phosphate or α-d-glucose 6-phosphate (25 mM) and 5 mM ZnCl_2_ for 62 and 4 min, respectively, prior to flash freezing in liquid nitrogen. Crystallographic data were collected using beamline ID29 at the European Synchrotron Radiation Facility in Grenoble, France. Diffraction images were processed with XDS^34^, and the integrated data were scaled with CCP4/Scala^35^.

### Structure determination and refinement

The crystal structure of the free enzyme and of the substrate and product complexes were determined by the molecular replacement program Mr. Bump^36^ method using the atomic coordinates taken from the structure of rabbit muscle phosphoglucomutase refined at 2.4 Å resolution^14^ (PDB deposition 3PMG). All structures were refined using phenix.refine within the PHENIX program package^37^. Several iterations of positional refinement in PHENIX interspersed with manual rebuilding in Coot^38^ resulted in final models with excellent geometries. Electron density maps of the ligands G1P and G6P were calculated using Polder OMIT maps^39^ as implemented in PHENIX, which excludes the bulk solvent around the omitted region and provides a better representation of the ligands in the active site. Data collection and refinement statistics are summarized in Table 1. Structure factors and coordinates have been deposited in the Protein Data Bank (PDB). The PDB accession numbers are listed in Table 1.

### Sequence collection and analysis

Sequences homologous to human PGM1 were obtained from the NCBI RefSeq and non-redundant protein databases^40,41^ with standard BLAST^42^ sequence searching. The sequences were carefully checked and curated employing the NCBI Gene database^41^ and linked resources, in particular RNA-Seq data from the short read archive (SRA) data resource^43^, as well as the Ensembl database^44^. Sequences were aligned with Muscle^45^, and the multiple sequence alignments were viewed and edited with Jalview^46^.

The sequence substitution model best fitting the protein data (segment corresponding to *PGM1* exons 2 to 11) was determined with ProtTest 3.4.2^47^. The best available model was the WAG empirical matrix^48^ with a discontinuous gamma distribution (Γ) for modelling rate heterogeneity over sites, *i.e*. WAG + Γ. Bayesian inference of phylogeny was determined with MrBayes 3.2.2^49^ with default priors and heating parameters (three heated Markov chain Monte Carlo chains and one cold). Two simultaneous and independent runs were carried out with sampling every 10 of 200 k generations until average standard deviation of split frequencies were below 0.001. Majority rule consensus tree topologies with branch lengths were calculated after discarding a burn-in of 50 k generations after which stationarity had been reached. The final potential scale reduction factors (PSRFs) were within 3×10^−3^ of 1.0 for all parameters.

Dendroscope 3.5.9^50^ was used for phylogenetic tree visualization. Structural disorder predictions were generated with DISOPRED3^51^ and PrDOS^52^. Sequence logos were generated with WebLogo 3.6 using an equiprobable background composition^53^. All protein structure illustrations were generated with PyMOL 2.2.2 from Schrödinger, LLC. Structural superpositioning of PGM full-length structures and domains, and calculation of Cα RMSD values, were performed with the PyMOL ‘align cycles=0’ function. The ‘angle_between_domains’ command from the PSICO PyMOL extension was used for comparing domain displacements between the different PGM structures. The PISA tool was used to examine putative multimerization of PGM1-2^30^.

## Supplementary information

Supplementary information.

## Data Availability

Structural data are available in the RCSB Protein Data Bank under the accession numbers 6SNP, 6SNO and 6SNQ. Other data are available from the corresponding author upon reasonable request.

## References

[1] Péanne R (2018). Congenital disorders of glycosylation (CDG): Quo vadis?. Eur. J. Med. Genet..

[2] Stojkovic T (2009). Muscle glycogenosis due to phosphoglucomutase 1 deficiency. N. Engl. J. Med..

[3] Tegtmeyer LC (2014). Multiple phenotypes in phosphoglucomutase 1 deficiency. N. Engl. J. Med..

[4] Wong SY-W (2016). Defining the phenotype and assessing severity in phosphoglucomutase-1 deficiency. J. Pediatr..

[5] Radenkovic S, Witters P, Morava E (2018). Central nervous involvement is common in PGM1-CDG. Mol. Genet. Metab..

[6] Radenkovic S (2019). The metabolic map into the pathomechanism and treatment of PGM1-CDG. Am. J. Hum. Genet..

[7] Stiers KM, Muenks AG, Beamer LJ (2017). Biology, mechanism, and structure of enzymes in the α-D-phosphohexomutase superfamily. Adv. Protein Chem. Struct. Biol..

[8] Beamer LJ (2015). Mutations in hereditary phosphoglucomutase 1 deficiency map to key regions of enzyme structure and function. J. Inherit. Metab. Dis..

[9] Muenks AG, Stiers KM, Beamer LJ (2017). Sequence-structure relationships, expression profiles, and disease-associated mutations in the paralogs of phosphoglucomutase 1. PLoS ONE.

[10] Cantu JM, Ibarra B (1982). Phosphoglucomutase: evidence for a new locus expressed in human milk. Science.

[11] Putt W (1993). Phosphoglucomutase 1: a gene with two promoters and a duplicated first exon. Biochem. J..

[12] Ray WJ, Burgner JW, Post CB (1990). Characterization of vanadate-based transition-state-analogue complexes of phosphoglucomutase by spectral and NMR techniques. Biochemistry.

[13] Ray WJ, Post CB, Liu Y, Rhyu GI (1993). Structural changes at the metal ion binding site during the phosphoglucomutase reaction. Biochemistry.

[14] Liu Y, Ray WJ, Baranidharan S (1997). Structure of rabbit muscle phosphoglucomutase refined at 2.4 Å resolution. Acta Crystallogr. Sect. D Biol. Crystallogr.

[15] Stiers KM, Kain BN, Graham AC, Beamer LJ (2016). Induced structural disorder as a molecular mechanism for enzyme dysfunction in phosphoglucomutase 1 deficiency. J. Mol. Biol..

[16] Stiers KM, Graham AC, Kain BN, Beamer LJ (2017). Asp263 missense variants perturb the active site of human phosphoglucomutase 1. FEBS J..

[17] Stiers KM, Beamer LJ (2018). A hotspot for disease-associated variants of human PGM1 is associated with impaired ligand binding and loop dynamics. Structure.

[18] Shackelford GS, Regni CA, Beamer LJ (2004). Evolutionary trace analysis of the α-D-phosphohexomutase superfamily. Protein Sci..

[19] Lee YS (1992). Purification, characterization, and molecular cloning of a 60-kDa phosphoprotein in rabbit skeletal sarcoplasmic reticulum which is an isoform of phosphoglucomutase. J. Biol. Chem..

[20] Whitehouse DB, Tomkins J, Lovegrove JU, Hopkinson DA, McMillan WO (1998). A phylogenetic approach to the identification of phosphoglucomutase genes. Mol. Biol. Evol..

[21] Brazeau MD, Friedman M (2015). The origin and early phylogenetic history of jawed vertebrates. Nature.

[22] Stiers KM, Beamer LJ (2018). Assessment and impacts of phosphorylation on protein flexibility of the α-D-phosphohexomutases. Methods Enzymol..

[23] Regni C, Naught L, Tipton PA, Beamer LJ (2004). Structural basis of diverse substrate recognition by the enzyme PMM/PGM from *P. aeruginosa*. Structure.

[24] Stiers KM (2019). Structural and dynamical description of the enzymatic reaction of a phosphohexomutase. Struct. Dyn..

[25] Naught LE, Tipton PA (2001). Kinetic mechanism and pH dependence of the kinetic parameters of *Pseudomonas aeruginosa* phosphomannomutase/phosphoglucomutase. Arch. Biochem. Biophys..

[26] Lynch M, Conery JS (2000). The evolutionary fate and consequences of duplicate genes. Science.

[27] Aguet F (2017). Genetic effects on gene expression across human tissues. Nature.

[28] Luebbering EK (2012). Conservation of functionally important global motions in an enzyme superfamily across varying quaternary structures. J. Mol. Biol..

[29] Mehra-Chaudhary R, Mick J, Tanner JJ, Henzl MT, Beamer LJ (2011). Crystal structure of a bacterial phosphoglucomutase, an enzyme involved in the virulence of multiple human pathogens. Proteins.

[30] Krissinel E, Henrick K (2007). Inference of macromolecular assemblies from crystalline state. J. Mol. Biol..

[31] Lee Y, Mehra-Chaudhary R, Furdui C, Beamer LJ (2013). Identification of an essential active-site residue in the α-D-phosphohexomutase enzyme superfamily. FEBS J..

[32] Lowry OH, Passonneau JV (1969). Phosphoglucomutase kinetics with the phosphates of fructose, glucose, mannose, ribose, and galactose. J. Biol. Chem..

[33] Regni C, Schramm AM, Beamer LJ (2006). The reaction of phosphohexomutase from *Pseudomonas aeruginosa*: structural insights into a simple processive enzyme. J. Biol. Chem..

[34] Kabsch W (2010). XDS. Acta Crystallogr. Sect. D Biol. Crystallogr.

[35] Collabortive computational project, number 4 (1994). The CCP4 suite: programs for protein crystallography. Acta Crystallogr. Sect. D Biol. Crystallogr.

[36] Keegan RM, Winn MD (2007). Automated search-model discovery and preparation for structure solution by molecular replacement. Acta Crystallogr. Sect. D Biol. Crystallogr.

[37] Adams PD (2010). PHENIX: a comprehensive Python-based system for macromolecular structure solution. Acta Crystallogr. Sect. D Biol. Crystallogr.

[38] Emsley P, Cowtan K (2004). Coot: Model-building tools for molecular graphics. Acta Crystallogr. Sect. D Biol. Crystallogr.

[39] Liebschner D (2017). Polder maps: improving OMIT maps by excluding bulk solvent. Acta Crystallogr. Sect. D Struct. Biol.

[40] O’Leary NA (2016). Reference sequence (RefSeq) database at NCBI: current status, taxonomic expansion, and functional annotation. Nucleic Acids Res..

[41] NCBI Resource Coordinators (2017). Database Resources of the National Center for Biotechnology Information. Nucleic Acids Res..

[42] Johnson M (2008). NCBI BLAST: a better web interface. Nucleic Acids Res..

[43] Kodama Y (2012). The sequence read archive: explosive growth of sequencing data. Nucleic Acids Res..

[44] Aken BL (2017). Ensembl 2017. Nucleic Acids Res..

[45] Edgar RC (2004). MUSCLE: multiple sequence alignment with high accuracy and high throughput. Nucleic Acids Res..

[46] Waterhouse AM, Procter JB, Martin DMA, Clamp M, Barton GJ (2009). Jalview Version 2 – a multiple sequence alignment editor and analysis workbench. Bioinformatics.

[47] Darriba D, Taboada GL, Doallo R, Posada D (2011). ProtTest 3: fast selection of best-fit models of protein evolution. Bioinformatics.

[48] Whelan S, Goldman N (2001). A general empirical model of protein evolution derived from multiple protein families using a maximum-likelihood approach. Mol. Biol. Evol.

[49] Ronquist F (2012). MrBayes 3.2: efficient Bayesian phylogenetic inference and model choice across a large model space. Syst. Biol..

[50] Huson DH, Scornavacca C (2012). Dendroscope 3: an interactive tool for rooted phylogenetic trees and networks. Syst. Biol.

[51] Jones DT, Cozzetto D (2015). DISOPRED3: precise disordered region predictions with annotated protein-binding activity. Bioinformatics.

[52] Ishida T, Kinoshita K (2007). PrDOS: prediction of disordered protein regions from amino acid sequence. Nucleic Acids Res..

[53] Crooks GE, Hon G, Chandonia JM, Brenner SE (2004). WebLogo: a sequence logo generator. Genome Res..

