# Supplementary material for: Structural basis for substrate and product recognition in human phosphoglucomutase-1 (PGM1) isoform 2, a member of the α-d-phosphohexomutase superfamily
Source: Sci Rep. 2020 Mar 27;10:5656. doi: 10.1038/s41598-020-62548-0 (PMC7101342; doi:10.1038/s41598-020-62548-0)
Supplement: Supplementary file 1 — Supplementary information. [file 41598_2020_62548_MOESM1_ESM.pdf]

Supplementary Information

Structural basis for substrate and product recognition in human phosphoglucomutase-1 (PGM1) isoform 2, a member of the α-D-phosphohexomutase superfamily

Paul Hoff Backe[1,2,†,*], Jon K. Laerdahl[1,3,†], Lene Svendsen Kittelsen[2], Bjørn Dalhus[1,2], Lars Mørkrid[2,4], and Magnar Bjørås[1,5,6]

From the [1]Department of Microbiology, Oslo University Hospital, Oslo, Norway; [2]Department of Medical Biochemistry, Institute for Clinical Medicine, University of Oslo, Oslo, Norway; [3]ELIXIR Norway, Department of Informatics, University of Oslo, Oslo, Norway; [4]Department of Medical Biochemistry, Oslo University Hospital, Oslo, Norway; [5]Department of Microbiology, Institute for Clinical Medicine, University of Oslo, Oslo, Norway; [6]Department of Clinical and Molecular Medicine, Norwegian University of Science and Technology (NTNU), Trondheim, Norway

†These authors wish it to be known that, in their opinion, the first two authors should be regarded as joint First Authors.

Figure S1. Multiple sequence alignment of human PGM1-1 and PGM1-2 with 96 orthologs, human PGM5 with 29 orthologs, as well as 18 homologs from invertebrate species

Figure S2. Secondary structure elements of PGM1-2

Figure S3. The N-terminal α-helical extension of PGM1-2 has poor packing with the rest of the protein

Figure S4. Structural disorder predictions for PGM1-2

Figure S5. Sequence logos show unique conserved segments and residues in PGM1-1 and PGM1-2

Figure S6. The current structures of PGM1-2 complexes have a more closed active site compared with recently published complex structures of *Xanthomonas citri* PMM/PGM

Table S1. Order of secondary structure elements in PGM1-2

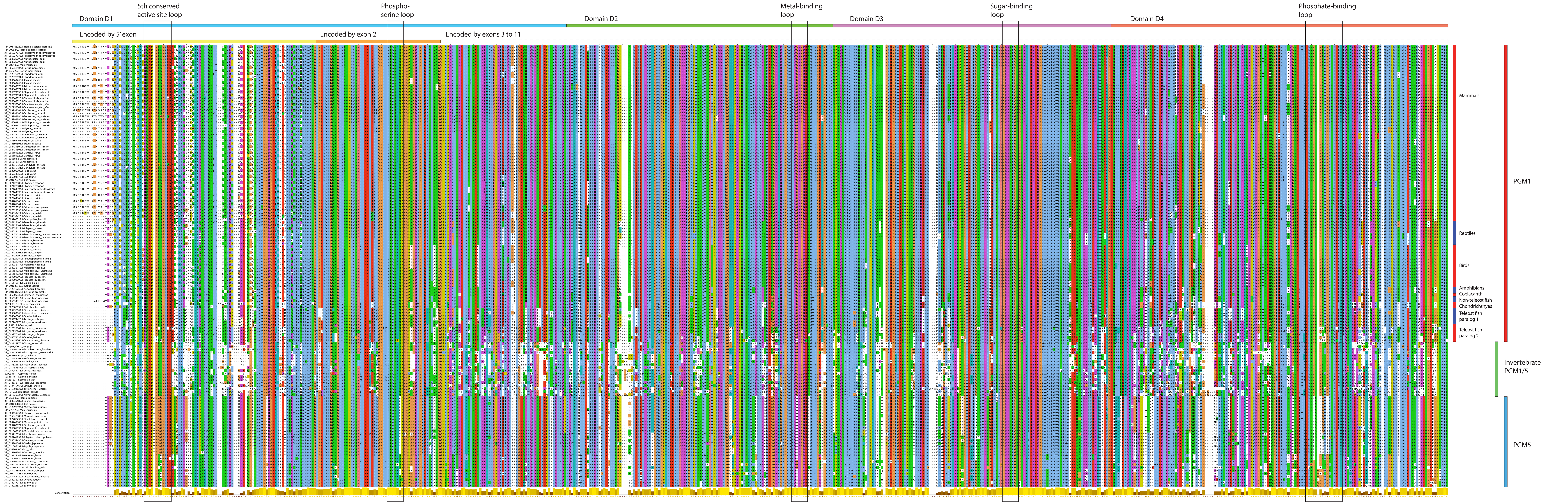

Figure S1. Multiple sequence alignment of human PGM1-1 and PGM1-2 (*i.e.* isoforms 1 and 2) with 96 orthologs (marked with red bar at right hand side), human PGM5 with 29 orthologs (blue bar), as well as 18 homologs from invertebrate species (green bar). The PGM1 sequences are marked according to vertebrate classes and main groups at the right. The yellow bar above the figure shows the segment encoded by the 5' exons in human PGM1 and PGM5, that is, exon 1-1 in PGM1-1, exon 1-2 in PGM1-2, and exon 1 in PGM5. The orange bar highlights the segment encoded by exon 2 in PGM1/5. The location of the four structural domains, D1 to D4, is shown above the alignment. The five boxes highlights the four previously described active site loops and the new active site loop discussed in the manuscript. The figure was prepared with Jalview (http://www.jalview.org).

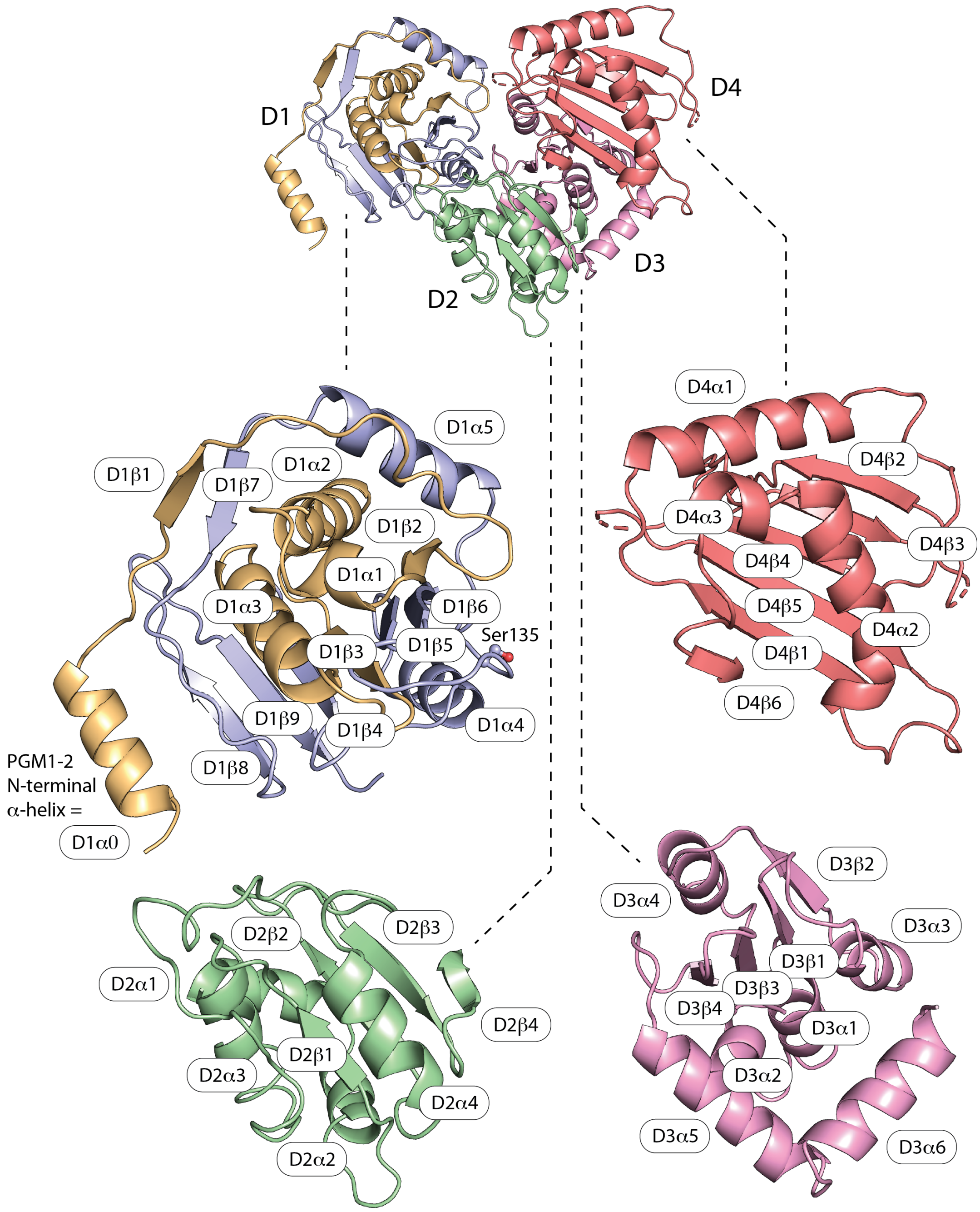

Figure S2. Secondary structure elements of PGM1-2. Human PGM1-2 is shown with identical orientation and coloring as in Fig. 2a (top), and annotated secondary structure elements, β-strands and α-helices (> 3 residues), are shown for the separated domains (bottom). The secondary structure elements and the corresponding residue ranges are given in Supplementary Table S1. Elements D1α0 to D1α3 are encoded by *PGM1* exon 1-1 (light orange, residues 1 to 100), unique to PGM1-2. Catalytic residue Ser135, located in the loop between D1β5 and D1β6, is shown with ball-and-stick representation.

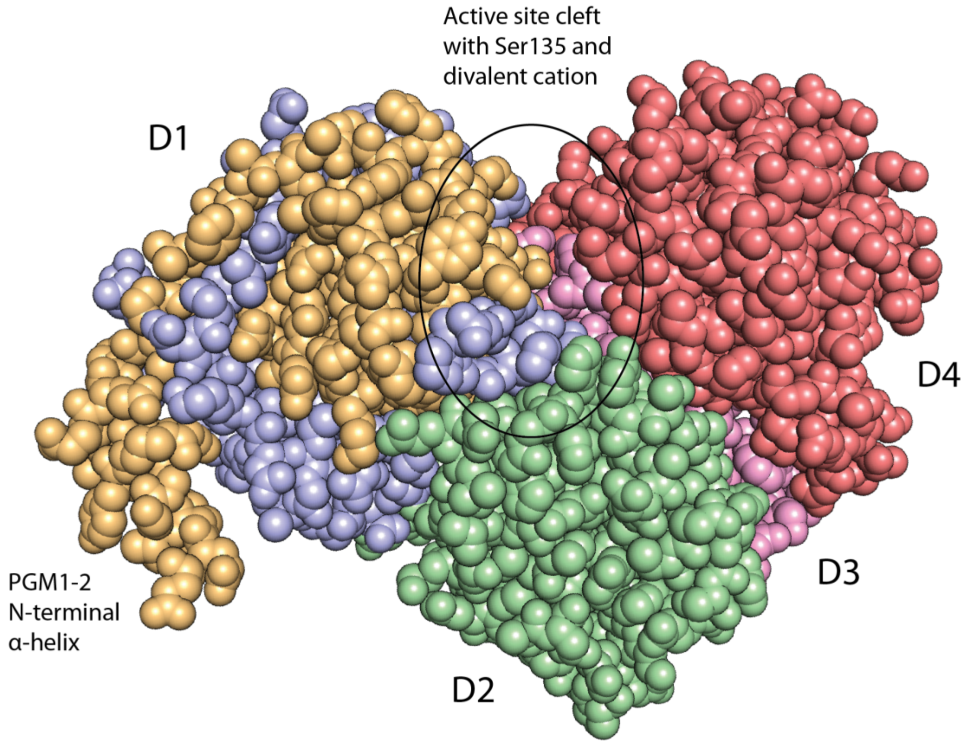

Figure S3. The N-terminal α-helical extension of PGM1-2 has poor packing with the rest of the protein. The PGM1-2 free protein is shown as a space-filling CPK calotte model with identical coloring as in Fig. 2a. Domains D4, D3, and D2 are shown in red, pink, and green, respectively. The PGM1-2 isoform specific exon 1-2 encoded segment is shown in light orange, while the rest of D1 is colored blue. The contact surface between the N-terminal helix (residues 5 to 16) and the rest of D1 is roughly 175 Å^2, calculated with the get_area command after addition of hydrogen atoms in PyMOL 2.2.2 from Schrödinger, LLC. In the crystal, the N-terminal helix interacts with two other nearby protein chains, with contact surfaces of approximately 475 Å^2 and 70 Å^2. There are no indications that these interactions between PGM1-2 chains occur in solution.

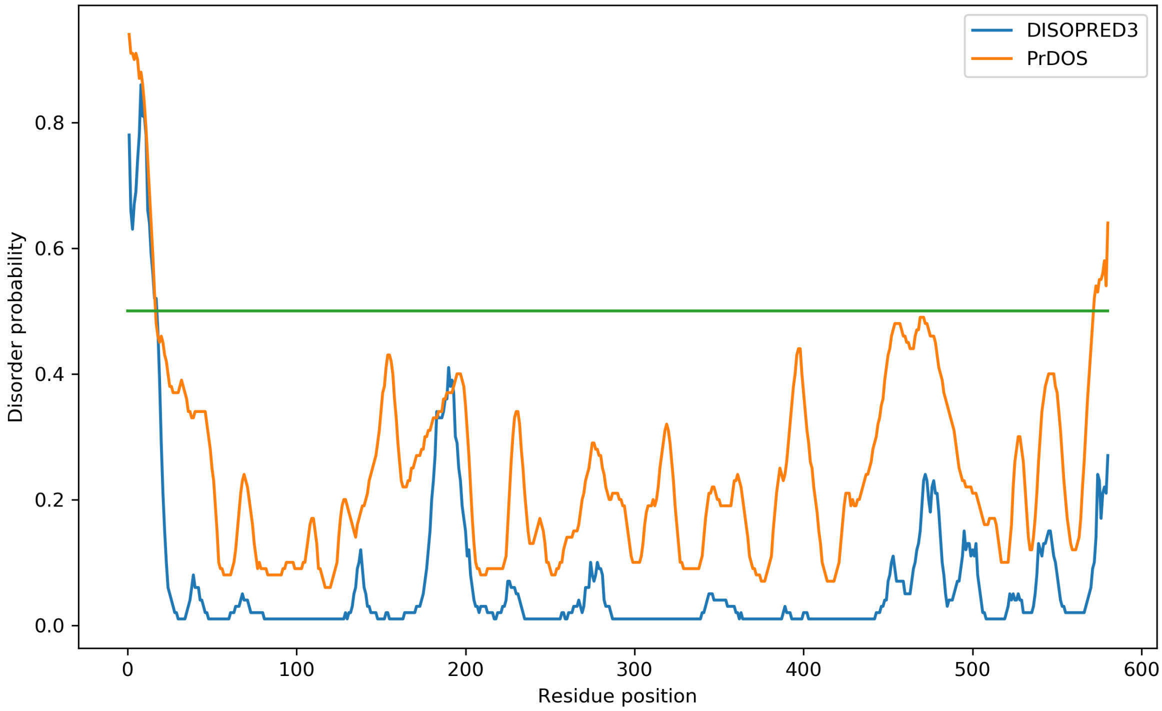

Figure S4. Structural disorder predictions for PGM1-2. The N-terminus of human PGM1-2 is predicted to be natively disordered by prediction software DISOPRED3 (residues 1 to 17) and PrDOS (residues 1 to 16), given a cutoff/threshold at 0.5 (green line). PrDOS also predicts the C-terminus to be disordered (residues 572 to 580), but with quite low probability.

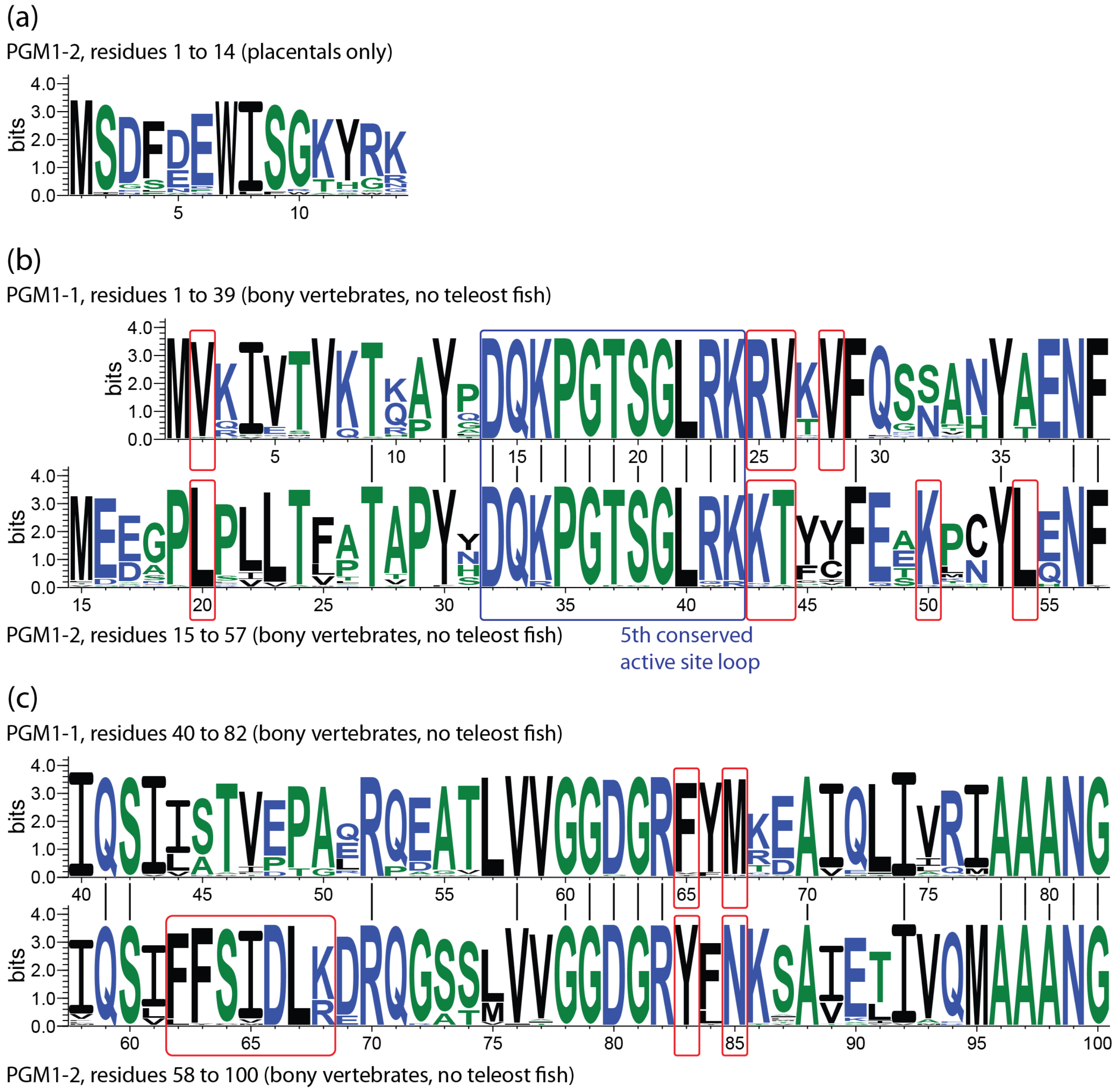

Figure S5. Sequence logos show unique conserved segments and residues in PGM1-1 and PGM1-2. (a) Logo generated from an alignment of the N-termini of 28 placental PGM1-2 orthologs (See Supplementary Fig. S1 for the sequences) shows overrepresentation of charged and aromatic residues and a complete conservation of human PGM1-2 Trp7. (b) Logo for PGM1-1 residues 1 to 39 (top) and the paralogous segment of PGM1-2 (bottom) generated from an alignment of 42 and 43 vertebrate homologs, respectively (See Supplementary Fig. S1 for the sequences). All Euteleostomi homologs in Supplementary Fig. S1 were included, except teleost fish, due to the gene duplication in these species. Black lines connect highly conserved paralogous residues. Red boxes highlight residues that are highly conserved in one isoform, but where this variant is very rare, or not found at all, in the other. (c) Logo for PGM1-1 residues 40 to 82 (top) and the paralogous segment of PGM1-2 residues 58 to 100 (bottom) generated in the same fashion and with the same annotation as in panel (b).

There are no insertions or deletions in the multiple sequence alignment of 85 vertebrate PGM1 sequences used for generating the panels (a) to (c). Consequently, stack numbering also corresponds directly to residue numbering in human PGM1-1 and PGM1-2. Stack heights indicate sequence conservation measured in bits, and the symbol heights reflect the relative frequency of the corresponding residues. An equiprobable background composition was used for logo generation.

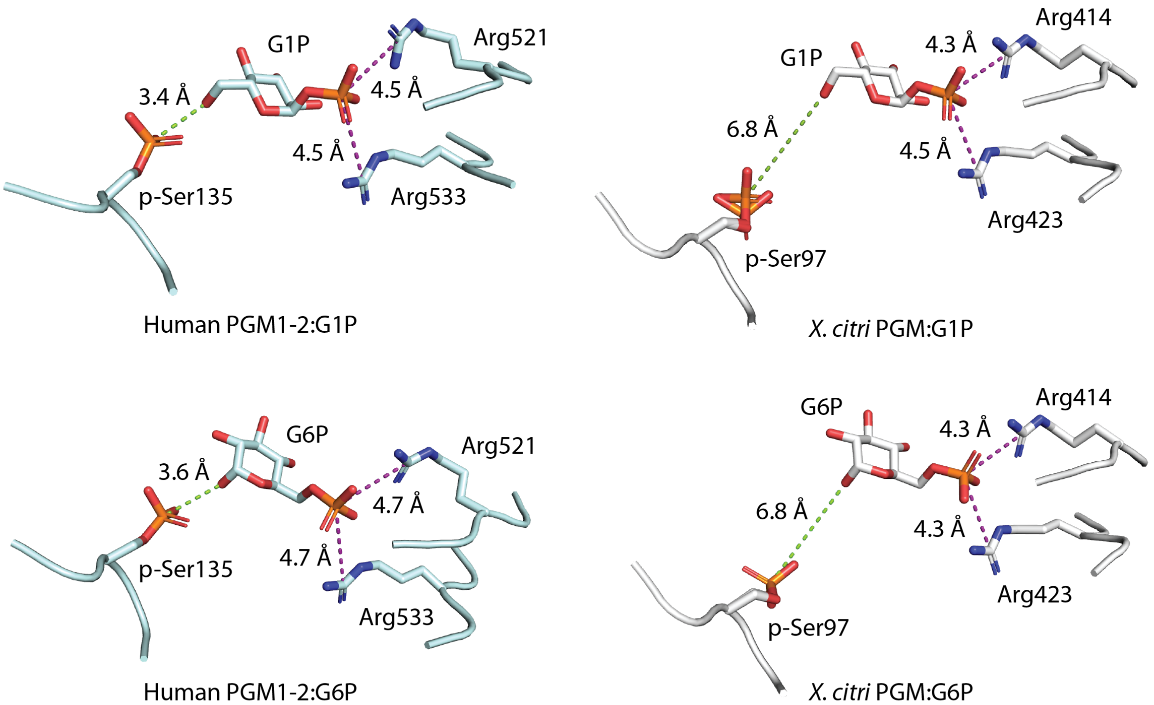

Figure S6. The current structures of PGM1-2 complexes have a more closed active site compared with recently published complex structures of *Xanthomonas citri* PMM/PGM. The phosphate-binding loops are interacting with the phosphate group of G1P (top panels) and G6P (bottom panels) with close to identical conformations for the two conserved Arg residues in human PGM1-2 (pale cyan, left hand panels, structures from current study, see also Fig. 5) and *X. citri* PMM/PGM (white, right hand panels, structures from PDB identifiers 6NNO and 6NNS (Stiers *et al.*, *Struct. Dyn.* **6**, 1 (2019)). The G1P and G6P hydroxyl groups involved in nucleophilic attack on active site phosphoserine (p-Ser) are located approximately 3.5 Å (green dotted line) from the P atoms of the phosphoryl groups in the PGM1-2 complexes (See also Figs. 5a and 5b). The corresponding distances in the *X. citri* PMM/PGM structures are longer than 6.5 Å, reflecting the much more open active site in these structures. The *X. citri* enzyme appears to belong to the PMM/PGM subgroup of PHMs although only PGM activity have been investigated experimentally (Stiers *et al.*, *Struct. Dyn.* **6**, 1 (2019)). The 6NNO and 6NNS structures have two alternative, but very similar, p-Ser conformations in the PDB files.

Table S1. Order of secondary structure elements in PGM1-2. See Supplementary Fig. S2 for location of secondary structure elements in the tertiary structure.

	Secondary structure element*	Residue range in PGM1-2**
Domain D1 (residues 1 – 209):		
	D1α0	7 – 17
	D1β1	23 – 26
	D1β2	40 – 43
	D1α1	44 – 48
	D1α2	53 – 64
	D1β3	74 – 79
	D1α3	85 – 98
	D1β4	103 – 107
	D1α4	114 – 124
	D1β5	128 – 132
	D1β6	144 – 150
	D1α5	159 – 171
	D1β7	174 – 177
	D1β8	189 – 193
	D1β9	202 – 207
Domain D2 (210 – 322):		
	D2α1	211 – 220
	D2α2	223 – 230
	D2β1	238 – 241
	D2α3	247 – 253
	D2α4	288 – 296
	D2β2	301 – 305
	D2β3	312 – 316
	D2β4	320 – 322
Domain D3 (323 – 439):		
	D3α1	324 – 333
	D3α2	339 – 344
	D3β1	349 – 352
	D3α3	358 – 365
	D3β2	370 – 373
	D3α4	377 – 385
	D3β3	391 – 394
	D3β4	398 – 401
	D3α5	409 – 423
	D3α6	427 – 438
Domain D4 (440 – 580):		
	D4β1	440 – 448
	D4α1	453 – 468
	D4β2	486 – 491
	D4β3	508 – 512
	D4β4	517 – 522
	D4β5	532 – 540
	D4α2	550 – 565
	D4α3	567 – 571
	D4β6	578 – 580

*Only α-helices longer than 3 residues have been annotated

**Subtract 18 to get PGM1-1 residue ranges
